# A novel malformation complex of bilateral and symmetric preaxial radial ray-thumb aplasia and lower limb defects with minimal facial dysmorphic features: a case report and literature review

**DOI:** 10.1186/1757-1626-1-271

**Published:** 2008-10-24

**Authors:** Ali Al Kaissi, Klaus Klaushofer, Alexander Krebs, Franz Grill

**Affiliations:** 1 Ludwig-Boltzmann Institute of Osteology at the Hanusch Hospital of WGKK and AUVA Trauma Centre Meidling, 4th Medical Department, Hanusch Hospital, Vienna, Austria; 2 Orthopaedic Hospital of Speising, Paediatric Department, Vienna, Austria

## Abstract

**Introduction:**

Radial hemimelia is a congenital abnormality characterised by the partial or complete absence of the radius. The longitudinal hemimelia indicates the absence of one or more bones along the preaxial (medial) or postaxial (lateral) side of the limb. Preaxial limb defects occurred more frequently with a combination of microtia, esophageal atresia, anorectal atresia, heart defects, unilateral kidney dysgenesis, and some axial skeletal defects. Postaxial acrofacial dysostoses are characterised by distinctive facies and postaxial limb deficiencies, involving the 5^th ^finger, metacarpal/ulnar/fibular/and metatarsal.

**Case presentation:**

The patient, an 8-year-old-boy with minimal craniofacial dysmorphic features but with profound upper limb defects of bilateral and symmetrical absence of the radius and the thumbs respectively. In addition, there was a unilateral tibio-fibular hypoplasia (hemimelia) associated with hypoplasia of the terminal phalanges and malsegmentation of the upper thoracic vertebrae, causing effectively the development of thoracic kyphosis.

**Conclusion:**

In the typical form of the preaxial acrofacial dysostosis, there are aberrations in the development of the first and second branchial arches and limb buds. The craniofacial dysmorphic features are characteristic such as micrognathia, zygomatic hypoplasia, cleft palate, and preaxial limb defects. Nager and de Reynier in 1948, who used the term acrofacial dysostosis (AFD) to distinguish the condition from mandibulofacial dysostosis. Neither the facial features nor the limb defects in our present patient appear to be absolutely typical with the previously reported cases of AFD. Our patient expands the phenotype of syndromic preaxial limb malformation complex. He might represent a new syndromic entity of mild naso-maxillary malformation in connection with axial and extra-axial malformation complex.

## Introduction

Pre and or/postaxial limb abnormalities are syndromic features in connection with AFD syndromes. The acrofacial dysostoses are heterogenous disorders combining varying severities of mandibulofacial dysostosis with pre and/or postaxial limb abnormalities. Malar hypoplasia and lower lid ectropion have been found in all patients. Micrognathia is the rule and this tends to improve with age. Cleft palate has been present in several cases [1-4]. Postaxial limb deficiencies include absence/hypoplasia/dysplasia of the fifth fingers in all limbs together with short forearms. Several syndromic entities have been described in the literature namely Miller syndrome, which must be differentiated from Treacher Collins and Nager syndromes [5,6]. The majority of patients with preaxial acrofacial dysostosis syndrome appear to represent sporadic occurrences. Nevertheless, substantial evidence supports an autosomal recessive pattern of inheritance and or dominant inheritance [3,4]. Limb defects can occur in connection with other forms of syndromic associations. Unilateral defects occurred with anomalies suggesting VACTERL association, facio-auriculo-radial anomaly, and CHILD'S syndrome [7-9]. Bilateral defects occurred more often in genetic or potentially genetic disorders such as ectrodactyly-ectodermal dysplasia-tibial aplasia, oro-mandibular-limb syndrome, and intercalary defects [10-12]. Our present patient presented with minimal facial features, but with profound limb defects of preaxial radial ray-thumb aplasia entity. Autosomal recessive pattern of inheritance was suggested in connection with parental consanguinity.

## Case presentation

**Figure 1:**
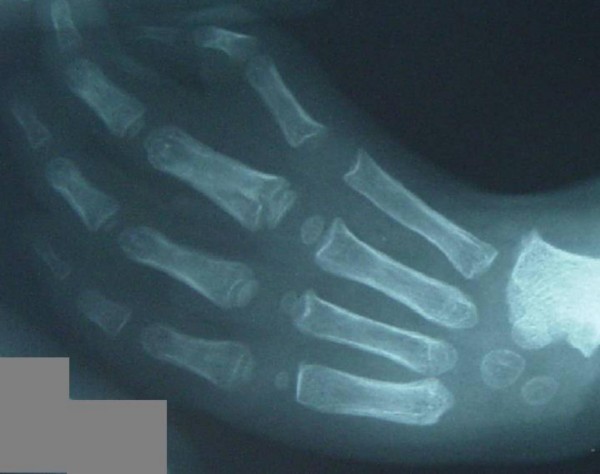
Anteroposterior radiograph of the hand showed the absent thumb.

**Figure 2:**
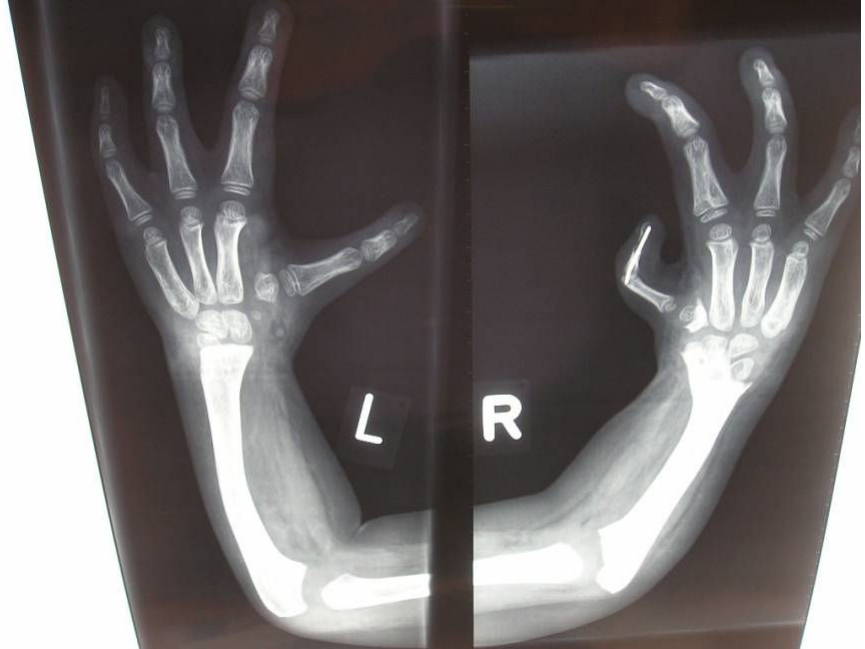
**Anteroposterior radiograph of the upper limbs after the reconstruction showed profound radial aplasia, metaphyseal cupping associated with hypoplastic/defective ossification of the carpal bones.** Pseudoepiphyses with proximally situated newly reconstructed thumb. The patient had reconstruction of the thumbs with pollizisation of the second finger. This means a rotation-osteotomy of the second finger to the thumb position with reconstruction of the commissural between the new reconstructed thumbs and the second finger, which was originally the third finger.

The boy was referred to the orthopaedic department because of multiple limb defects for clinical assessment and treatment. He was a product of uneventful gestation. At birth his growth parameters were around the 10 Th percentile. The mother was a 26-year-old gravida 2 abortus 0 married to a 28-year-old related man (first cousin). Pregnancy history was negative for exposure to drugs or teratogens. Family history was unremarkable. At birth a near normal facies but with bilaterally dysplastic ears, associated with profound shortening of the upper limbs and a unilateral lower limb defects were present. Bilateral and symmetric radial ray and thumb aplasia associated with lower limb inequality secondary to a unilateral tibiofibular hypoplasia and hypoplasia of the terminal phalanges was the most prominent major abnormalities. He underwent a series of orthopaedic interventions. Primarily, the thumbs were absent on both sides (fig 1). The patient had reconstruction of the thumbs with pollizisation of the second finger. This means a rotation-osteotomy of the second finger to the thumb position with reconstruction of the commissural between the new reconstructed thumbs and the second finger, which was originally the third finger. Anteroposterior radiograph of the upper limbs after the reconstruction showed profound radial aplasia, metaphyseal cupping associated with hypoplastic/defective ossification of the carpal bones. Pseudoepiphyses with proximally situated newly reconstructed thumb (fig 2)

At the age of 4 years Ilizarov technique was performed to correct the lower limb defects (Distraction osteogenesis, this refers to the induction of new bone between bone surfaces that are pulled apart in a gradual, controlled manner). His subsequent course of development particularly the motor and the fine movements have been of significant retardation because of the profound limb defects. No medical history of serious illnesses.

Examination at the age of eight years, showed a boy with normal intelligence with normal speech, hearing and vision. Craniofacially, his face was minimally dysmorphic. Bilateral dysplastic ears, and extension of his hair to the lateral part of the forehead were present. Clinically the nose looks normal but radiographically there was mild nasomaxillary hypoplasia. The spine showed significant thoracic kyphosis.

**Figure 3:**
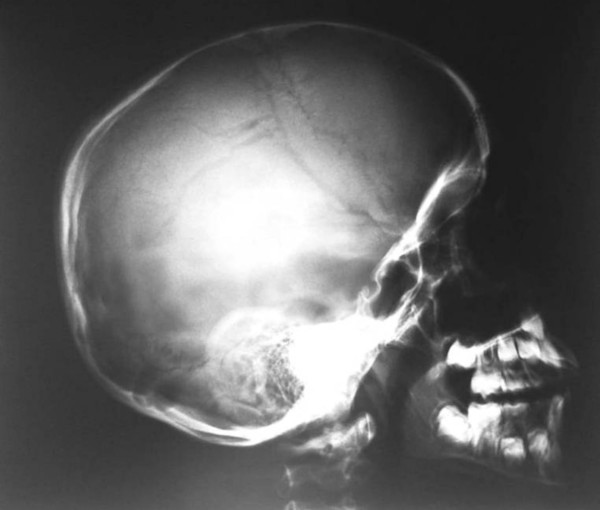
Lateral skull radiograph showed diffuse skull-base sclerosis associated with J-shaped cella turcica and a large sphenoid sinus, multiple patent sutures, mild nasomaxillary/mandibular hypoplasia and odontoid hypoplasia.

**Figure 4:**
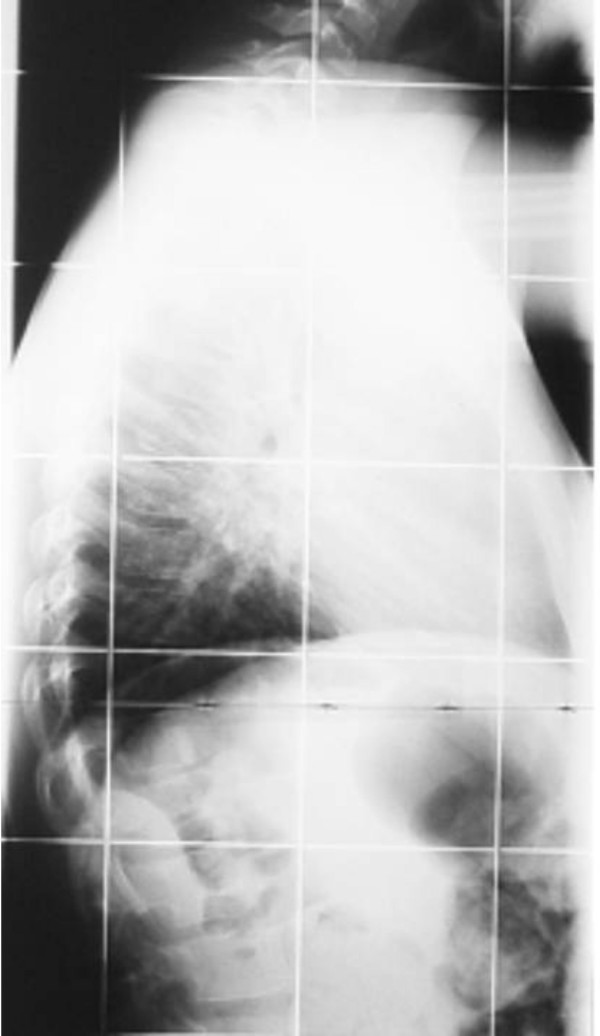
**Lateral spine radiograph showed significant thoracic kyphosis secondary to malsegmentation of the upper thoracic vertebrae associated with scalloping of the posterior-end plates of the lumbar vertebrae.** There was exaggerating lumbar lordosis associated with thoracic kyphosis.

**Figure 5:**
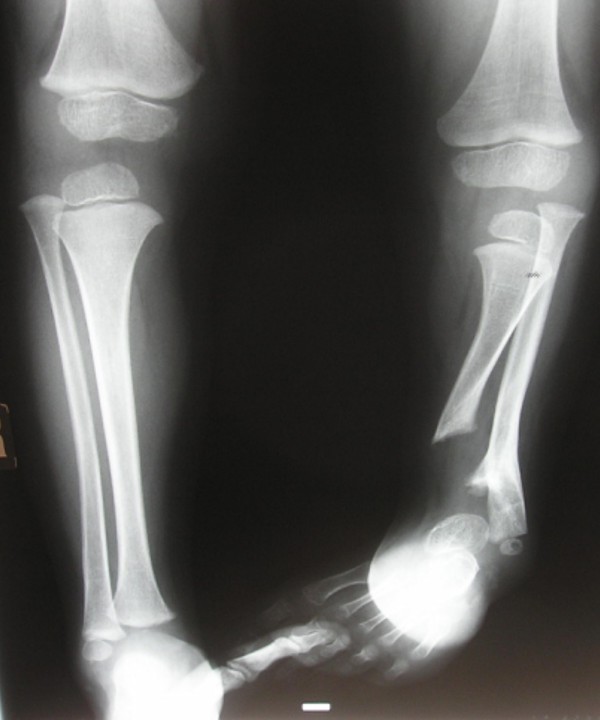
**Anteroposterior radiograph of the lower limbs showed profound unilateral tibiofibular hypoplasia with subsequent development of ball and socket ankle joint secondary to fibular-tarsal dysplasia.** Dysplasia of the tarsal and the metatarsals and hypoplasia of the terminal phalanges were evident. There was relative left tibial hypoplasia.

**Figure 6:**
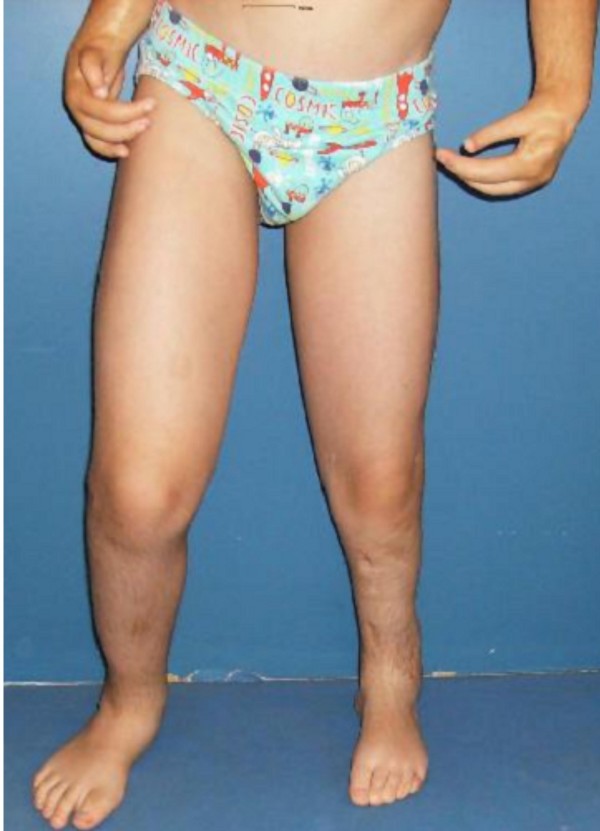
**Ilizarov technique has been successfully performed for the lower limb deformity. **The discrepancy in the muscle bulk of the lower limbs was secondary to disuse atrophy.

He had a short stature of -2 SD, profound and bilateral mesomelic shortening of the upper limbs (absent radii) associated with oligodactyly (bilateral absence of the first fingers) and partial syndactyly. A unilateral tibiofibular hypoplasia was evident with subsequent development of ball and socket (ankle joint) deformity in connection with absent talus. Mild hypoplasia of the terminal phalanges of the deformed lower limb was present. Laboratory studies showed normal white and red blood cell and platelet counts, normal calcium, phosphorus, and alkaline phosphatase levels. Urine aminoacids and mucopolysaccharides were normal, and he had a normal karyotype. No specific genetic testing has been designated for this child. Renal ultrasound and echocardiodoppler were normal. On the bases of skeletal survey, the lateral skull radiograph showed unusual skull-base sclerosis associated with J-shaped cella turcica and a large sphenoid sinus, multiple patent sutures, mild nasomaxillary/mandibular hypoplasia and odontoid hypoplasia (fig 3). Lateral spine radiograph showed malsegmentation of the upper thoracic vertebrae associated with scalloping of the posterior-end plates of the lumbar vertebrae. There was exaggerating lumbar lordosis associated with thoracic kyphosis (fig 4). Anteroposterior radiograph of the lower limbs showed profound unilateral tibiofibular hypoplasia with subsequent development of ball and socket ankle joint secondary to fibular-tarsal dysplasia. The right tibia showed relative hypoplasia. Dysplasia of the tarsal and the metatarsals and hypoplasia of the terminal phalanges were evident (fig 5). Ilizarov technique has been successfully performed for the lower limb deformity. The discrepancy in the muscle bulk of the lower limbs was secondary to disuse atrophy (fig 6).

## Discussion

Limb defects associated with other malformation complex (whether it is major/minor) require prompt characterisation. There have been a number of syndromic malformation complex in connection with preaxial/postaxial limb defects. Nager acrofacial dysostosis is an oromandibular hypogenesis syndrome with associated limb abnormalities. Although it shares some phenotypic features with Treacher-Collins syndrome, it is recognized as a separate disorder. The facial defects in Nager syndrome include an antimongoloid eye slant, malar and mandibular hypoplasia and small, malformed or low-set ears. The thumbs may be absent or hypoplastic and there may be hypoplasia of the radius with radioulnar synostosis. Limb defects, particularly preaxial anomalies, are of diagnostic significance in Nager syndrome, and serve to differentiate this condition from mandibulofacial dysostosis. The radial defects are believed only to occur with concurrent agenesis of the thumb. Defects of the lower extremity also have been described. Heterogeneity of apparently non-syndromal acrofacial dysostosis of both types (Nager and Treacher-Collins) is powerful support for the hypothesis that the AFDs are polytopic field defects arising during balstogenesis [1-6]. Thompson et al., [13] reported a case of preaxial acrofacial dysostosis with tetralogy of Fallot. Fryns et al., [14] reported an adult with features of the condition who had mild mental handicap. Neurological examination revealed hemiparesis of the left arm and severe spastic diplegia of the lower legs with general hyperreflexia. Herrmann et al., [15] reported conductive hearing loss was noted in 90% of his cases. Inheritance is uncertain because there is evidence for both autosomal dominant and recessive inheritance in different families. Guttmacher [16] reported a father, son and daughter with unusual hand and foot abnormalities, and hypospadias in the males. There was hypoplasia of the thumbs and halluces with postaxial polydactyly. There was also limitation of movement of the interphalangeal joints of the thumbs in the daughter and prominent vascularity on the posterior of her right forearm. The second toes had one phalanx and absent nails. Lettice et al., [17] studied a three-year-old girl with bilateral duplication of a triphalangeal thumb and triplication of the great toe. She had a de novo t(5,7)(q11, q36) translocation. The breakpoints on 7q36 lay within the LMBR1 gene about 1 Mb away from SHH. A similar translocation in the same intron of Lmbr1 in the mouse was also associated with preaxial polydactyly and genetic analysis in the mouse suggested that the translocation directly interrupted a cis acting regulator of SHH. None of the above mentioned entities entails similar malformation complex as seen in our present patient.

## In summary

Limb defects are a diverse collection of conditions, each developing through a different prenatal process, probably with different underlying causes. Proper classification is critical. Surgery is often needed in upper limb defects as well as for lower limb defects to straighten and stabilize the legs for prosthesis fitting. Finally we wish to stress that the profound limb deformities in our present patient were noteworthy. The extent of these malformations was not compatible with any previously reported entities of syndromic and non-syndromic forms of preaxial malformation complex.

## Abbreviations

SD: Standard deviation; AFDs: Acrofacial dysostoses; LMBR1: limb region 1 homolog (mouse); SHH: Sonic hedgehog gene and protein.

## Competing interests

The authors declare that they have no competing interests.

## Authors' contributions

All of the authors were involved in the clinico-radiographic assessment and finalising the paper. All authors have red and approved the final version of the paper.

## Consent

Written informed consent was obtained from the parents for the purpose of publication of the manuscript and figures of their child. A copy of the written consent is available for review by the editor-in-Chief of this journal.
